# Holter features to detect coronary artery spasm in ANOCA patients: A pilot study

**DOI:** 10.1016/j.ijcha.2026.101884

**Published:** 2026-02-12

**Authors:** Diantha JM Schipaanboord, Caïa Crooijmans, Nicole S Erler, Peter David Faasse, Tijn PJ Jansen, Timo Nijkamp, Saskia ZH Rittersma, Tim P van de Hoef, Pim van der Harst, Aukelien C Dimitriu-Leen, Peter Damman, Suzette E Elias-Smale, René van Es, N Charlotte Onland-Moret, Hester M den Ruijter, on behalf of the IMPRESS consortium

**Affiliations:** a Laboratory of Experimental Cardiology, University Medical Center Utrecht, Utrecht University, Utrecht, the Netherlands; b Department of Cardiology, Radboud University Medical Center, Nijmegen, the Netherlands; c Julius Center for Health Sciences and Primary Care, University Medical Center Utrecht, Utrecht University, Utrecht, the Netherlands; d Department of Cardiology, Division Heart and Lungs, University Medical Center Utrecht, Utrecht University, Utrecht, the Netherlands

**Keywords:** Holter, ECG, Coronary artery spasm, Ischemia, Circadian, ANOCA

## Abstract

**Background:**

Coronary artery spasm (CAS), which can be epicardial and/or microvascular, is highly prevalent in patients with angina and non-obstructive coronary artery disease (ANOCA) undergoing coronary function testing (CFT). The CFT is invasive and limits larger diagnostics studies. We studied if Holter monitoring with symptom tracking identifies Holter-based CAS features with diagnostic potential in ANOCA patients.

**Methods:**

42 ANOCA patients (88% female) were recruited in the UMCU-IMPRESS pilot study and wore a 12-lead Holter device for 2–7 days prior to CFT, with simultaneous symptom tracking. We compared symptoms and Holter-ECG characteristics between patients with and without CAS and calculated diagnostic measures for CAS using several thresholds for ischemia-related parameters.

**Results:**

33 Patients were diagnosed with CAS (79%). These patients more often had ≥ 1 min of ST depression in total per day compared to patients without CAS (≥0.035 mV: 88% vs 44%, p = 0.013; ≥0.040 mV: 73% vs 33%, p = 0.049), but discriminative ability was limited (AUC (95% CI): 0.65 (0.48–0.68)). Furthermore, patients with CAS had periods of lower heart rates and longer PQ and QT times than patients without CAS, most evident at night and early morning.

**Conclusions:**

Patients with CAS more often demonstrated at least one minute of ST depression in total per day and exhibited periods of lower heart rates and longer PQ times mainly during the night and early morning compared to patients without CAS. Although discriminative ability was limited, we show that Holter monitoring may reveal signals in CAS patients, substantiating the need of large (AI-based) studies.

## Graphical abstract

**Figure:**
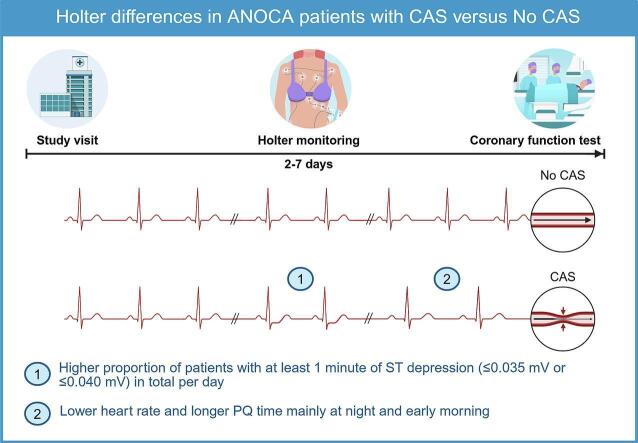
Illustration of the study flow of ANOCA patients who participated in the UMCU-IMPRESS pilot study and wore a Holter for 2-7 days between a study visit and coronary function testing (CFT), with the aim of discovering Holter-based measures with diagnostic potential for coronary artery spasm (CAS). The CFT resulted in a diagnosis of CAS or the absence thereof (No CAS). Patients with CAS had (1) a higher proportion of patients with at least one minute of ST depression per day, and (2) periods of lower heart rates and longer PQ times (most evident at night or early morning) than patients without CAS. Created in https://BioRender.com. Holter monitoring and coronary function test illustration: Colin Cramm.

## 1 Introduction

Women constitute the majority (50–70%) of patients suffering from angina and non-obstructive coronary artery disease (ANOCA) [1], [2] and most have underlying coronary vasomotor dysfunction (CVDys).[3] While CVDys can be attributed to coronary microvascular dysfunction (CMD), more than 90% of the patients have coronary artery spasm (CAS), either in isolation or in combination with CMD.[4] CAS temporarily narrows the epicardial coronary arteries or microvascular arterioles, restricting myocardial blood flow, triggering symptoms such as angina or dyspnea.[5].

The current gold standard to diagnose CAS is pharmacological vasospasm provocation testing during invasive coronary function testing (CFT), which relies on the presence of ischemic changes on the electrocardiogram (ECG) during the procedure. This procedure typically involves intracoronary acetylcholine infusion [2], [6], [7], which is generally considered to be safe but may lead to life-threatening adverse reactions in up to 1% of patients.[8], [9], [10] For final diagnosis, COVADIS criteria additionally require that ischemic changes coincide with symptomatic episodes. [11].

Pharmacologically induced coronary artery spasm during provocation testing may not accurately mirror real-life spasm dynamics, and the invasive nature and associated risks and costs make spasm provocation testing not ideal for a broad population of ANOCA patients, thereby increasing the threshold for testing and contributing to underdiagnosis. As episodes of symptoms may represent transient ischemic events detectable as changes on an ECG, using ECG detection in the diagnostic path seems logical. However, patients often do not experience symptoms or show ECG changes during clinical testing. Therefore, the 2024 ESC guidelines for the management of chronic coronary symptoms recommends to consider ambulatory 12-lead ECG monitoring in patients suspected of CAS. [2] However, evidence for this recommendation is lacking as it relies on only two small studies (n = 22–26), neither of which employed acetylcholine provocation testing for spasm diagnosis, which is the current golden standard. Nor did their study design fit a diagnostic study including all ANOCA patients referred for spasm diagnosis, but only included patients already diagnosed with CAS.[12], [13] Hence, a substantial evidence gap persists concerning the effectiveness of Holter monitoring in capturing ECG abnormalities in patients with CAS.

Although Holter monitoring potentially offers a non-invasive, real-world alternative for the detection of CAS, uncertainty remains regarding which ECG changes are indicative of CAS. In this pilot study, we aim to identify Holter-based measures, including ischemic changes and conduction times, with diagnostic potential for CAS that deserve further studies.

## 2 Methods

### 2.1 Study population and design

The UMCU-IMPRESS pilot study is a diagnostic cross-sectional study from the University Medical Center Utrecht (UMC Utrecht) and Radboud University Medical Center (Radboudumc), the Netherlands. Between November 2021 and January 2025 patients with anginal symptoms despite medical therapy, who were referred for CFT and had no obstructive CAD as established with cardiac CT or coronary angiography (<50% stenosis, fractional flow reserve > 0.80 or an instant wave-free ratio > 0.89), were invited to participate. The Medical Ethical Review Committee of the UMC Utrecht has approved the study and all patients provided informed consent.

Every participant had to visit the UMC Utrecht two to seven days prior to CFT. This study visit comprised multiple assessments, including a questionnaire and body surface mapping in rest and during exercise. In addition, this visit included venous blood sampling for participants of the Radboudumc and could comprise a measurement with the handheld miniECG [14]. At the end of the visit, patients received a 12-lead Holter for ECG recording at home, which was returned on the day of the CFT. On this day arterial blood was sampled for participants of the UMC Utrecht.

### 2.2 Questionnaire

Patients completed a structured questionnaire assessing demographics, risk factors, medical history, and medication use. Given that patients with ANOCA are known to experience symptoms for an extended period before receiving a definitive diagnosis, the questionnaire also covered symptom characteristics, such as symptom type, onset, duration, and frequency, enabling self-reported retrospective assessment of symptom burden and diagnostic delay. [15] To indicate their symptom type, patients could choose multiple from the following four predefined categories: 1) chest pain, pressure or tightness, 2) shortness of breath or difficulty breathing, 3) fatigue or lack of energy, and 4) other symptoms.

### 2.3 Holter recording

The 12-lead Holter device (SenseLink, TeMeCo B.V.) was applied using ten skin electrodes placed during the study visit at the UMC Utrecht. We instructed the patients to continue their usual daily activities and to wear the Holter until at least the evening prior to the day of CFT. The Holter continuously measured the 12-lead ECG at a sampling frequency of 200 Hz, except when the battery needed to be replaced. They had to disconnect the Holter only before taking a shower or bath and reapply the device with new electrodes on the same locations on the skin afterwards. To assess symptom incidence and type, symptoms were prospectively recorded while ANOCA patients wore the Holter device. Patients were instructed to press a button on the device and report symptom characteristics such as type (similar categories as in the questionnaire) and duration in an event diary in case of recognizable symptoms. Patients returned the Holter and event diary on the day of CFT prior to the procedure. The raw 12-lead ECG data were extracted from the device by the study team.

### 2.4 Medication use

During Holter recording, participants continued their prescribed medication as usual. However, in preparation for the CFT, patients paused the intake of long-acting anti-anginal medication and other vasoactive substances 1–2 days prior to CFT to ensure that vasoreactivity during the spasm provocation test was not influenced by medication. This medication pause overlapped with the final days of Holter recording.

### 2.5 Coronary function testing

Participants underwent CFT at the UMC Utrecht or Radboudumc as part of their regular diagnostic work-up. CFT was performed according to the previously described protocol.[16] CFT started with diagnostic coronary angiography to rule out obstructive CAD followed by spasm provocation testing and assessment of microvascular function.

Spasm provocation testing was conducted by subsequent intracoronary infusion of 2, 20, 100, and 200  μg acetylcholine, with angiography following each dose. If > 90% epicardial vasoconstriction occurred, higher doses were withheld.[16] CAS was diagnosed according to the COVADIS criteria, which entails having anginal symptoms and ischemic ECG changes in combination with > 90% vasoconstriction on the angiogram (epicardial CAS) or < 90% vasoconstriction (microvascular CAS) assessed after each acetylcholine dose.[11] If clinically indicated, an acetylcholine rechallenge was performed by readministering the same dose that had previously induced spasm approximately three minutes after intracoronary nitroglycerin to assess spasm reducibility.

The interventional cardiologists assessed the presence of CMD with the bolus thermodilution method using adenosine or continuous thermodilution method, as described previously. CMD was diagnosed in case of a coronary flow reserve (CFR) < 2.0 with bolus thermodilution or absolute CFR < 2.5 with continuous thermodilution.[2].

### 2.6 Holter characteristics

To analyze the Holter data, we used Galvo 12 L (V1.0, Cordys Analytics), that included a segmentation algorithm developed by the UMC Utrecht and Cordys Analytics. Galvo 12 L identifies heartbeat locations and their classification, as well as the on– and offsets of P-waves, QRS complexes, ST-segments, the isoelectric line, and noise artifacts. From its output, we derived median heart rate and conduction times per 10-seconds, and the standard deviation of the R-R intervals of sinus rhythm beats (SDNN: a time-domain measure of heart rate variability) per five-minute intervals. Galvo 12 L also calculates a median beat per lead per 10-second segment, which we used to calculate ST deviation and T-wave amplitude deviations relative to each patient's own baseline median beat matched by heart rate. A detailed description of the preprocessing steps and calculation of the used measures are in Supplementary Method 1.

### 2.7 Main Holter assessments

Holter monitoring primarily aims to detect ischemia in patients with CAS, aligning with both the COVADIS criteria(11) and ESC guideline(2) recommendation. Additionally, Holter monitoring can provide insight into 24-hour variation in conduction times, as circadian rhythms are known to affect cardiac electrophysiology throughout the day.[17] Finally, Holter monitoring with symptom tracking enables analysis of 10-second resting ECGs under standardized conditions to minimize variability from time of day, heart rate, symptoms, and medication. To uncover ECG patterns that may not be detected using a single method, we included three distinct Holter assessments: (1) ischemia assessment, (2) 24-hour assessment, and (3) 10-second assessment.

To evaluate ischemia we used the complete Holter recording per patient and used an exploratory approach to investigate promising ischemia-related parameters and thresholds. For each ST-T-related parameter (ST deviation, ST elevation, ST depression, deviation in minimum T-wave amplitude or deviation in maximum T-wave amplitude), ischemia was defined using a range of cut-off values from 0-0.5 mV (e.g., ST-depression ≥0.1 mV) in at least two contiguous leads for at least 30 s. For each ST-T-related parameter and cut-off value, we divided the total ischemia duration by the total recording duration, to account for variability in recording duration across patients, and calculated the proportion of patients that had ≥1 min of ischemia in total per day. Using the ST-T-related parameter(s) and cut-off value(s) that showed to be promising to differentiate between patients with and without CAS, we calculated the percentage of acute symptoms (related to chest pain or dyspnea) that were concurrent with ischemic events (within ± 30 min of symptom onset). To explore circadian differences, we selected 24 h of Holter recording (12 PM-12 PM, first and last full day) for each patient. For the last assessment, we selected 10-seconds of Holter data (heart rate close to 75 bpm) during a symptom-free period between 06:30 and 08:30 AM preferably on the day prior to CFT, or, if not feasible, on the second day prior to CFT for each patient.

### 2.8 Statistical analysis

We present baseline characteristics for all patients stratified by CAS status after CFT. Continuous variables are presented as mean ± SD or median [Q1-Q3]. Categorical variables are presented as counts (%).

We compared the time (in years) from symptom onset to study inclusion (median [Q1–Q3]) and the presence of four symptom types (chest pain, pressure or tightness, shortness of breath or difficulty breathing, fatigue or lack of energy and other symptoms) between patients with and without CAS, based on data obtained with the questionnaire during the study visit. Symptom presence was reported as counts (%) and compared using the Chi-squared test between the groups.

Furthermore, we tested whether the total number of reported symptoms per day during Holter monitoring (from event diary and/or Holter button) and the type of reported symptoms during monitoring differed between patients with and without CAS using the Mann-Whitney *U* test.

First, we compared and plotted the proportion of patients with ≥1 min of daily ischemia in total (y-axis) across varying ST-T-related cut-off values (x-axis) between patients with and without CAS, with 95% CIs calculated using the Wilson score method. Differences were tested per measure and cut-off value using the fisher’s exact test. To adhere to the COVADIS criteria, we also compared the percentage of symptoms concurrent with ischemia between the groups (i.e. within a 30 min time frame). For ST-T measures with significant group differences or the combined measure (criteria: having symptoms concurrent with ischemia), we calculated the Area Under the Curve (AUC), sensitivity, specificity, PPV, and NPV (all with 95% CI). AUC confidence intervals were derived via bootstrapping, using 1000 bootstrap samples generated by resampling with replacement.

Next, we compared and plotted the heart rate, conduction times and SDNN per 3-hour interval (as median with 2.5th-97.5th percentile) over a 24-hour period for patients with and without CAS and tested differences between the groups using either an unpaired *T*-test or Mann-Whitney *U* test, where appropriate. For Holter measures that showed a significant difference between patients with and without CAS at specific 3-hour time intervals, we assessed their effect size in a logistic regression model, both unadjusted and adjusted for age, sex, beta-blocker use, and the use of calcium channel blockers known to influence heart rate.

Finally, for the 10-second Holter assessment, we compared heart rate and conduction times (mean ± SD or median [Q1-Q3]) and tested the conduction times between patients with and without CAS using the unpaired *T*-test or Mann-Whitney *U* test, where appropriate.

Analyses of baseline characteristics and symptom characteristics of the questionnaire were conducted using R (version 4.1.3). All Holter related analyses (symptoms and ECG) were performed using Python (version 3.8.10). A p-value < 0.05 was considered statistically significant. Given the exploratory nature of the analyses, findings should be interpreted as hypothesis-generating.

## 3 Results

### 3.1 Clinical characteristics

Between November 2021 and January 2025, 47 patients were included. Five were excluded because of a history of obstructive CAD treated with PCI (n = 2), a new obstructive CAD diagnosis at CFT (n = 2) or coronary artery dissection at CFT (n = 1). Therefore, 42 patients remained available for analysis. ANOCA patients included in this study were approximately 60 years of age (SD ± 8), mostly female (88%) and were -on average- slightly overweight (BMI: 26.0 [23.5–29.3]). They had a stenosis degree between 0 and 50% and a RFR > 0.89 and/or FFR > 0.80 (Supplementary Table 1). CFT resulted in a CAS diagnosis in 33 of the 42 ANOCA patients (79%), including four patients with combined CAS and CMD (12%). Among patients with CAS (n = 33), 48% had isolated microvascular spasm, 15% had both epicardial and microvascular spasm and 36% had epicardial spasm. In four of the latter patients, no acetylcholine re-challenge was performed, and therefore the presence of co-existing microvascular spasm could not be assessed. Of the nine patients without CAS, four had isolated CMD (44%).

Patients with and without CAS were comparable in age, BMI and sex (Table 1). Cardiovascular risk factors were highly prevalent in all patients, except for diabetes and current smoking. Hypertension was notably less common among patients with CAS compared to those without CAS (27% vs 67%, respectively). Furthermore, almost half of patients with CAS (48%) had a history of migraine compared to 22% in patients without CAS. In general, ANOCA patients used a considerable amount of anti-anginal medication, anti-platelets and cholesterol-lowering medication (Table 1) which did not notably differ between patients with and without CAS.

**Table 1:** Patient characteristics stratified by CAS diagnosis.

	No CAS (n = 9)	CAS (n = 33)
Age, years	63 ± 8	60 ± 8
Female, n (%)	8 (89%)	29 (88%)
Body mass index, kg/m^2^	26.7 [25.0 – 31.4]	26.0 [22.0 – 28.6]
Cardiovascular risk factors		
Hypertension, n (%)	6 (67%)	9 (27%)
Dyslipidemia, n (%)	3 (33%)	19 (58%)
Diabetes, n (%)	1 (11%)	3 (9%)
Current smoker, n (%)	1 (11%)	4 (12%)
Former smoker, n (%)	3 (33%)	15 (45%)
Alcohol use, n (%)	7 (78%)	19 (58%)
Stress, n (%)	3 (33%)	19 (58%)
Positive family history, n (%)	3 (33%)	10 (30%)
Other risk factors		
Migraine (history/current), n (%)	2 (22%)	16 (48%)
Raynaud’s disease, n (%)	1 (11%)	4 (12%)
Female-specific risk factors		
Women with pregnancy history, n (%)	6 (75%)	27 (93%)
Pregnancies, n	2.0 [2.0–2.8]	2.0 [2.0–3.0]
At least one miscarriage or premature birth, n (%)	2 (25%)	8 (28%)
Gestational complications, n (%)	1 (13%)	6 (21%)
Menopause ≤ 40 years, n (%)	0 (0%)	4 (14%)
Medical history		
Fibromyalgia, n (%)	1 (11%)	5 (15%)
Asthma, n (%)	2 (22%)	6 (18%)
Reduced thyroid function, n (%)	1 (11%)	6 (18%)
Auto-immune disease, n (%)	1 (11%)	6 (18%)
Cardiovascular history		
Supraventricular tachycardia, n (%)	1 (11%)	5 (15%)
Bundle branch block, n (%)	0 (0%)	2 (6%)
Valvular disease, n (%)	2 (22%)	5 (15%)
Vascular related diseases, n (%)	2 (22%)	3 (9%)
Medication use		
Beta blockers, n (%)	2 (22%)	7 (21%)
Calcium channel blockers, n (%)	5 (56%)	24 (73%)
Short-acting nitrates, n (%)	5 (56%)	23 (70%)
Long-acting nitrates, n (%)	4 (44%)	8 (24%)
Nicorandil, n (%)	0 (0%)	5 (15%)
Antiplatelets, n (%)	6 (67%)	17 (52%)
Anti-hypertensives, n (%)	2 (22%)	5 (15%)
Cholesterol lowering, n (%)	4 (44%)	20 (61%)

CAS = Coronary artery spasm.

### 3.2 Symptom characteristics − questionnaire

To understand the duration as well as the prevailing symptoms in the years before, we asked our study participants about their symptoms in a questionnaire. We observed that participants had endured symptoms for several years with a median duration of 2 [1], [2], [3], [4], [5] years. Furthermore, chest pain, pressure or tightness was present in almost all ANOCA patients (93%; Table 2). Although not statistically significant, patients diagnosed with CAS more often reported shortness of breath or difficulty breathing compared to patients without CAS (82% vs 44%, respectively, p = 0.07).

**Table 2:** Symptom characteristics as reported in the questionnaire during the study visit stratified by CAS diagnosis.

	No CAS (n = 9)	CAS (n = 33)	p-value
Time difference between onset of symptoms and study inclusion, years	2.0 [1.0 – 4.0]	2.0 [1.0 – 5.0]	
Symptom type			
Chest pain, pressure or tightness	9 (100%)	30 (91%)	0.83
Shortness of breath or difficulty breathing	4 (44%)	27 (82%)	0.07
Fatigue or lack of energy	6 (67%)	25 (76%)	0.90
Other symptoms	4 (44%)	13 (39%)	1.00

Time differences are noted as median [Q1-Q3]; symptom types as counts (%). CAS = Coronary artery spasm.

### 3.3 Symptom characteristics – Holter

**Fig. 1:**
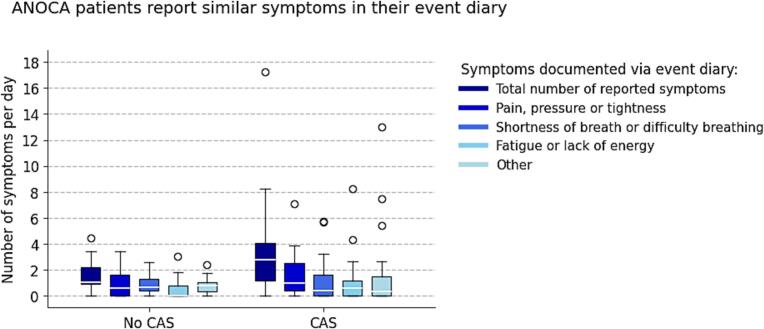
**Number of symptoms per day and per symptom type reported in the event diary during Holter monitoring for patients with and without CAS.** CAS = Coronary artery spasm.

The median Holter wear time was 3.6 [2.9–5.2] days (CAS: 3.9 [3.0–5.2] days; No CAS: 3.1 [2.3–4.9] days). Despite instructions, discrepancies existed between symptoms reported in the event diary and via the Holter’s event-marking button. ANOCA patients reported a median of 1.9 [1.0–3.8] and 1.4 [0.8–3.4] symptoms per day via the diary and button, respectively, which did not always overlap. Combining both methods, patients reported a median of 2.9 [1.3–4.4] symptoms per day, which was similar between patients with and without CAS (3.0 [1.4–4.9] vs. 2.1 [0.9–3.5] symptoms/day; p = 0.28; Supplementary Fig. 1). Characterization of symptom types was based on the event diary data, which mirrored the four symptom categories as used in the questionnaire. Their daily frequencies did not differ significantly between the groups (Fig. 1).

### 3.4 Holter assessment – Ischemia

**Fig. 2:**
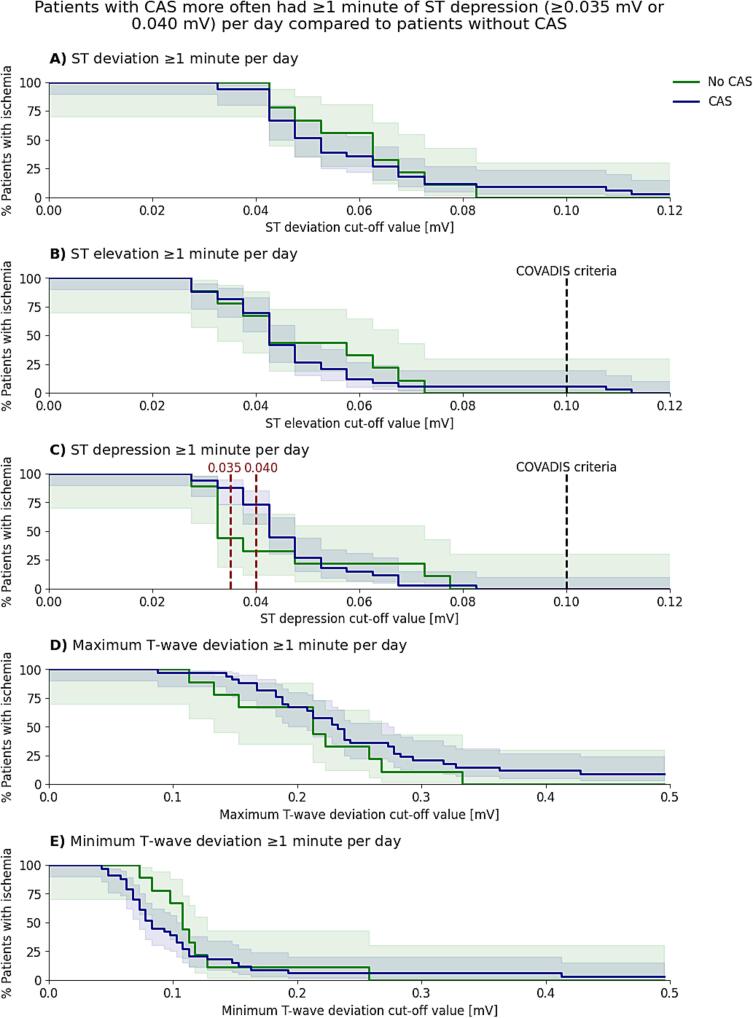
**Proportions with 95% confidence interval of patients with ≥1 min per day of A) ST deviation, B) ST elevation, C) ST depression, D) deviation in minimum T-wave, E) deviation in maximum T-wave, for different thresholds.** CAS = Coronary artery spasm. A red dashed line represents a p-value < 0.05. A black dashed line shows the cut-off threshold of the COVADIS criteria [11]. (For interpretation of the references to colour in this figure legend, the reader is referred to the web version of this article.)

**Fig. 3:**
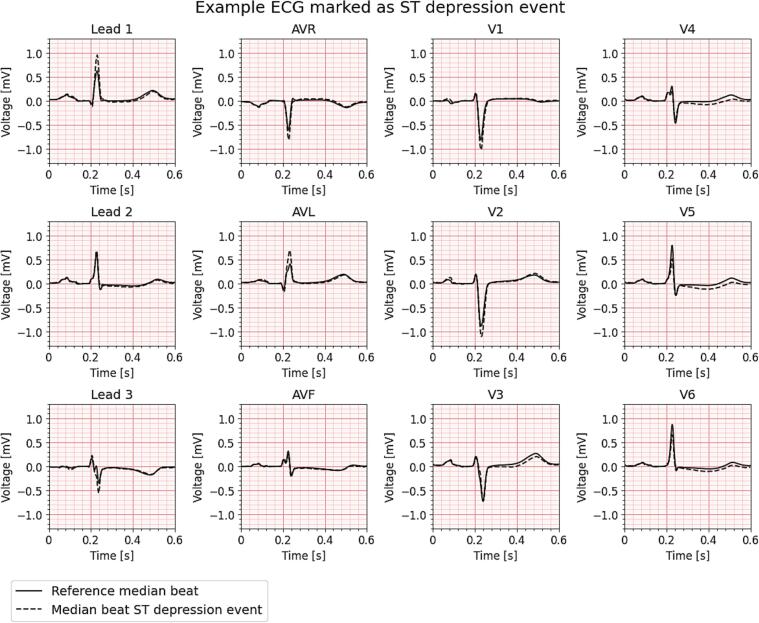
Example 12-lead median ECG beat from a patient diagnosed with coronary artery spasm, showing a detected ST-segment depression event in the lateral leads using a threshold of ≥0.035 mV (dashed line) versus the patient’s baseline median beat with corresponding heart rate (solid line).

To assess ischemia detection, we analysed the proportion of patients with and without CAS in whom ischemia could be detected, using different ischemia-based measures (ST-segment and T-wave related) and thresholds ranging from zero to 0.5 mV (Fig. 2). Statistically significant differences between patients with and without CAS occurred only at ST depression thresholds of ≥0.035 mV and ≥0.040 mV. At the ≥0.035 mV threshold, significantly more patients with CAS had ≥1 min of ST depression daily compared to patients without CAS (88% vs 44%; p = 0.013). At a higher threshold of ≥0.040 mV, this difference remained statistically significant, with 73% of patients with CAS and 33% of patients without CAS meeting this criterion (p = 0.049). No statistically significant differences were observed between the groups for any of the other ST-T parameters. As the 1–2 days of medication-free period prior to CFT may influence the incidence of ST-segment depression, we performed a time-dependent analysis and visually inspected that there were no time-dependent differences in ST-segment depression occurrence (Supplementary Fig. 2). An example ECG illustrating the detection of an ST depression event using a ≥0.035 mV threshold in a patient diagnosed with CAS is shown in Fig. 3.

The area under the curve (AUC = 0.65; 95% CI: 0.48–0.68) indicated limited discriminative ability (Table 3) for ≥ 1 min daily ST depression. Although the positive predictive value (PPV) was high for both ST depression thresholds (≥0.035 mV and ≥0.040 mV), negative predictive value (NPV) was low (Table 3). In addition, the sensitivity for CAS was high for ST depression of ≥0.035 mV and moderate for ST depression ≥0.040 mV but the specificity was low for both thresholds (Table 3).

**Table 3:** Preliminary diagnostic accuracy of Holter-based ischemia detection of CAS at different cut-off points for the definition of an ST depression either defined solely on ECG at any point in time and for ST depression concurrent with symptoms.

	Sensitivity, % (95% CI)	Specificity, % (95% CI)	PPV, % (95% CI)	NPV, % (95% CI)
≥1 min of daily ST depression (AUC = 0.65 (95% CI: 0.48–0.68)), criteria:				
≥0.035 mV	0.88 (0.73,0.95)	0.56 (0.27–0.81)	0.88 (0.73–0.95)	0.56 (0.27–0.81)
≥0.040 mV	0.73 (0.56–0.85)	0.67 (0.35–0.88)	0.89 (0.72–0.96)	0.40 (0.20–0.64)
≥1 symptom concurrent with ST depression (AUC = 0.49 (95% CI: 0.36–0.52)), criteria:				
≥0.035 mV	0.55 (0.38–0.70)	0.78 (0.45–0.94)	0.90 (0.70–0.97)	0.32 (0.16–0.53)
≥0.040 mV	0.36 (0.22–0.53)	0.89 (0.57–0.98)	0.92 (0.67–0.99)	0.28 (0.15–0.46)

AUC = Area under the receiver operating characteristic curve; CAS = Coronary artery spasm; NPV = Negative predictive value; PPV = Positive predictive value.

**Fig. 4:**
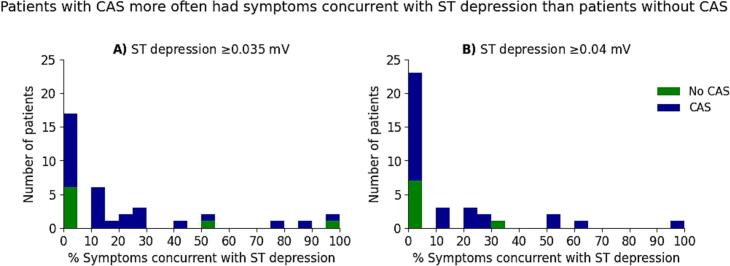
**Stacked histograms showing the percentage of symptoms (related to chest pain, pressure or tightness or shortness of breath or having difficulty breathing) occurring concurrently with ischemia on the full Holter recording.** Ischemia on the Holter was defined as **A**) ST depression ≥0.035 mV or **B**) ST depression ≥0.040 mV, within 30 min before or after the onset of symptoms for patients with CAS (blue) and without CAS (green). CAS = Coronary artery spasm. (For interpretation of the references to colour in this figure legend, the reader is referred to the web version of this article.)

To adhere to the COVADIS criteria, we further evaluated the proportion of acute symptoms – chest pain, pressure or tightness or shortness of breath or having difficulty breathing –whose onset linked to changes in the ECG, defined as the occurrence of a symptom within 30 min before or after ST depression ≥0.035 mV (Fig. 4A) or ST depression ≥0.040 mV (Fig. 4B). Six patients did not report any symptoms (CAS: n = 5, No CAS: n = 1) and were therefore excluded from this analysis. 64% Of the patients with CAS and symptoms (n = 18) and 25% of patients without CAS and symptoms (n = 2, of which one was diagnosed with CMD; p = 0.10) had at least one symptom concurrent with ST depression ≥0.035 mV and with ST depression of ≥0.040 mV this was 43% vs 13%, respectively (p = 0.21) (Fig. 4). The median percentage of symptoms concurrent with ST depression of ≥0.035 mV and ≥0.040 mV on the Holter was 14% [0–25%] and 0% [0–20%] for patients with CAS, respectively, and 0% [0–13%] and 0% [0–0%] for patients without CAS, respectively (Fig. 4).

We tested whether the presence of at least one symptom concurrent with ST depression would be able to distinguish patients with CAS from those without CAS, including for these analyses the patients without any symptoms. Having symptoms concurrent with ischemia was not predictive for the detection of CAS (AUC = 0.49 [95% CI: 0.36–0.52]). The presence of at least one symptom concurrent with ischemia demonstrated for both ST depression thresholds again high PPV and low NPV, but yielded moderate-to-high specificity and low sensitivity (Table 3).

### 3.5 Holter assessment – 24-hour recordings

**Fig. 5:**
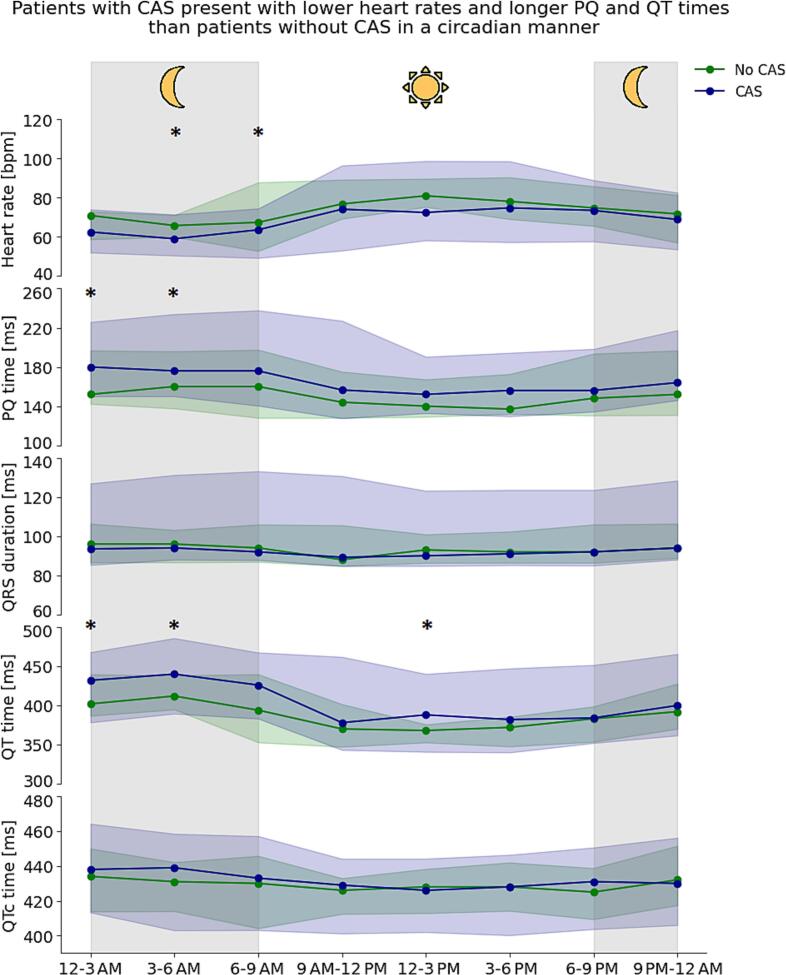
**Median conduction times per three hours over the last full day between patients with CAS (blue) and without CAS (green).** The shaded colours represent the 2.5th to 97.5th percentile. The number of patients per group and type of statistical test (parametric or non-parametric) used per characteristic and timepoint is detailed in Supplementary Table 2A. The * represents a p-value < 0.05. (For interpretation of the references to colour in this figure legend, the reader is referred to the web version of this article.)

During 24-hours of the last full day, as expected, circadian patterns were observed in heart rate and QT time with higher heart rates and shorter QT times during the day, and the reverse at night (Fig. 5). Patients with CAS had lower heart rates at all time points compared to patients without CAS, but this difference was larger and became statistically significant during the night and early morning (3–6 AM: 60.0 ± 6.5 bpm vs 65.2 ± 3.8 bpm, p = 0.03; 6–9 AM: 62.8 ± 7.4 bpm vs 69.8 ± 11.3 bpm, p = 0.04).

Although PQ and QT are dependent on heart rate, we investigated the differences in these intervals between patients with and without CAS as we hypothesized these may be independently influenced by circadian rhythm apart from heart rate. The QT times of patients with CAS were longer compared to patients without CAS, and again these differences were larger and statistically significant the night (12–3 AM: 429.5 ± 28.2 ms vs 408.7 ± 16.7 ms (p = 0.04); 3–6 AM: 438.0 ± 27.1 ms vs 413.3 ± 14.0 ms (p = 0.01)) and in the afternoon (12–3 PM: 386.3 ± 28.2 ms vs 365.9 ± 8.4 ms (p = 0.001)). However, when correcting the QT for heart rate (QTc), these differences were no longer present. PQ time also followed a circadian pattern, more pronounced in patients with CAS. The PQ time was longer in patients with CAS compared to patients without CAS, which again became stronger and statistically significant at night (12–3 AM: 180 [161–192] ms vs 152 [144–164] ms, p = 0.01; 3–6 AM: 176 [164–195] ms vs 160 [144–170] ms, p = 0.03).

The effect size of heart rate on CAS during 3–6 AM and 6–9 AM, as well as PQ time on CAS during 12–3 AM and 3–6 AM, remained similar or in some models even became slightly stronger after including age, sex and treatment for beta blockers or calcium channel blockers in the models.

Sensitivity analyses where we only included the heart rate and conduction times over the first full day or last full day in patients with and without CAS yielded reasonably similar results, with statistically significantly lower heart rates and longer PQ and QT times during the night (Supplementary Fig. 3). We did not observe significant differences in the SDNN per 3h over the last full day (Supplementary Fig. 4) between the groups.

### 3.6 Holter assessment – 10-second recordings

None of the conduction times obtained from 10-second recordings differed between patients with and without CAS (Table 4). We observed prolonged QTc times in 15 patients with CAS (45%) and in two patients without CAS (22%).

**Table 4:** Conduction times of 10-second Holter recordings.

	No CAS (n = 9)	CAS (n = 33)	P-value
Heart rate, bpm	75.0 [75.0–75.4]	75.0 [74.8–75.0]	
PQ time, ms	153 ± 23	166 ± 19	0.09
QRS duration, ms	96 [91–96]	91 [88–96]	0.38
QT time, ms	394 ± 15	405 ± 21	0.15
QTc time Bazett, ms	440 ± 17	451 ± 22	0.19

Values are noted as mean ± SD, or median [Q1–Q3]. CAS = Coronary artery spasm.

## 4 Discussion

In this pilot study, we aimed to identify Holter-based ECG features capable of distinguishing ANOCA patients with CAS by prospectively analysing 12-lead Holter recordings combined with symptom tracking in the days preceding CFT. We show that ANOCA patients with CAS more often had ≥ 1 min of ST depression (≥0.035 mV or ≥0.040 mV) per day compared to patients without CAS. The predictive value was however modest. Additionally, patients with CAS exhibited periods of lower heart rates and longer PQ and QT times compared to patients without CAS over the course of 24h, particularly during the night and early morning (12 AM to 9 AM).

The COVADIS criteria recommend a cut-off value of 0.1 mV for ST elevation and depression to detect transient ischemic ECG changes [11]. Our findings, including significant differences only at lower ST depression thresholds (≥0.035 mV and ≥0.040  mV) and the absence of differences in ST elevation, suggest that ischemic ECG changes in CAS patients may be more subtle than previously recognized. Frequent transient spasms in patients with CAS might precondition the heart, reducing ischemic susceptibility.[18] However, the lack of findings near the 0.1 mV threshold may also reflect our methodology, which relied on deviations from heart rate-matched baseline beats instead of the J-point elevation or depression at specific moments in time or due to the ischemia threshold of ≥1 min total per day. ST depressions of 0.035 mV and 0.040 mV are difficult to visually assess in clinical practice. However, the strength of automated ECG analysis lies in its ability to detect subtle changes that are difficult or impossible to identify reliably by eye. Furthermore, we cannot definitively conclude that the micro ST-segment depression observed in our study represents myocardial ischemia, as there are no differences between groups in universally accepted criteria for myocardial ischemia (i.e., ST segment depression or elevation >0.1 mV). We therefore consider the observed micro-ST depression as exploratory. The 0.1 mV cut-off defined by the COVADIS criteria should still be regarded as the primary clinical reference.

We hypothesized that ischemic changes would occur exclusively in patients with CAS and might be missed due to a limited observation period or the absence of symptoms leading to high PPV and specificity. Indeed, ≥1 min of ST depression (≥0.035 mV or ≥0.040  mV) in total daily showed a high PPV, but also identified ischemia in patients without CAS, resulting in low specificity. This finding may be attributed to CMD, undiagnosed CAS in patients with borderline CFT results, or potential measurement inaccuracies. Combining acute symptoms with ST depression slightly improved the specificity and PPV, but reduced the sensitivity and NPV, partly attributed to asymptomatic patients during Holter monitoring. The diagnostic value therefore remains modest. As our aim was to discover Holter-based measures with diagnostic potential for CAS, future research could consider extending Holter monitoring until (acute) symptoms occur.

The lower heart rates and longer PQ intervals in patients with CAS, especially at night and early morning, align with the finding that CAS mainly occurs at night and early in the morning [19]. While heart rate naturally declines at night, the significantly lower rates in patients with CAS may reflect a circadian mechanism. Differences in QT time between the groups can be explained by heart rate. Both PQ and QT times are heart rate-dependent, with QT being more strongly influenced. [20], [21] Since SDNN – reflecting total autonomic influence on heart rate – did not differ between the groups, autonomic nervous system differences between groups seem less likely. The cause of lower median heart rates in patients with CAS therefore remains unclear.

Beyond heart rate, longer PQ intervals in patients with CAS may reflect underlying endothelial dysfunction. Prior research suggested that a longer PR interval, even within the conventionally normal range, is independently linked to endothelial dysfunction and greater arterial stiffness in healthy individuals without atherosclerotic disease.[22] This can potentially also explain the higher PQ time observed in patients with CAS in this study, given endothelial dysfunction is one of the mechanisms proposed to contribute to CAS.[23], [24].

This study had some limitations that should be considered. First, this study was designed as a pilot study to investigate potentially diagnostic signals on Holter ECG for the diagnosis of CAS. Consequently, sample size was low. In addition, due to the high prevalence of CAS diagnosis in patients undergoing CFT [4], the group of patients without CAS was small, which further limited the power of this study. Due to the limited power and multiple comparisons, results should be interpreted with caution. The ischemia-related ECG patterns identified were not distinctive enough to serve as diagnostic markers at this stage. Nonetheless, our study did result in interesting non-invasive diagnostic measures that deserve further study. Given the results of our study, detecting the observed effect (AUC = 0.65) with a confidence interval width of 0.10 and a CAS prevalence of 80%, would require a sample size of 627 patients.[25] However, any further diagnostic study would need to demonstrate at least moderate accuracy (AUC ≥ 0.70) to justify further development.

In both groups, some patients were diagnosed with CMD, but the percentage of patients with CMD was higher in patients without CAS than with CAS (44% vs. 12%), potentially affecting results, as ECG changes like QTc prolongation are reported for patients with CMD.[26], [27] We did not exclude patients with CMD, as overlapping phenotypes are common and reflect the patient population in which the Holter will be applied best.

Patient-reported symptoms were incorporated into our Holter analyses, but did not improve the diagnostic accuracy for CAS. This may relate to their subjective nature and the inter-patient variability in both the experience (e.g. severity) and the reporting of symptoms. Inconsistencies between event diary entries and button presses also comprised data quality and completeness. A digital diary with built-in reminders and additional support could improve data accuracy and adherence. Additionally, symptoms like fatigue were commonly reported but often lacked clear on– and offset and persisted throughout the day, challenging the Holter analyses. Consequently, we focused on acute symptoms (chest pain and dyspnea-related), which may account for the limited overlap between symptoms and ST depression.

Another limitation is the variability in Holter recording duration across patients, resulting in varying data volumes used per patient for the ischemia analyses. In patients with short recordings, we may have failed to capture symptoms and ischemic events. Nonetheless, given a median of 2.9 (IQR: 1.3–4.4) symptoms in total per day and a minimum monitoring duration of two days, the likelihood of missing symptoms is considerably low.

Holter monitoring is susceptible to noise, with motion artefacts and loose electrodes making parts of the recording unusable. Additionally, interruptions due to showering or battery depletion further contributes to data loss. In our study, on average around 80% of the total recording was usable, which is quite high, showing that these recordings were successfully conducted. We further found that the equipment fulfilled the required safety and performance expectations.

Finally, medication use may have influenced the results. Patients however paused the intake of long-acting anti-anginal medication 1–2 days before the CFT. These days were included in the 10-seconds and 24-hour Holter assessment (last full day). We did not observe major conduction time differences between the first and last full day (Fig. 2 and Supplementary Fig. 3) for the 24-hour assessment and the effect size of heart rate and PQ time on CAS remained similar after including treatment for beta blockers or calcium channel blockers in the models. Hence, the influence of medication use appears to be negligible, but cannot be ruled out. Accordingly, future large-scale diagnostic studies should take medication use into account.

This pilot study provided a unique opportunity to investigate patient-reported symptoms and Holter-derived ECG patterns in ANOCA patients with and without CAS in the days preceding CFT. The findings warrant further research into the added value of Holter monitoring for CAS diagnosis in this population. Additionally, a large diagnostic study could enable the opportunity to examine ECG differences between patients with and without CMD and the development of statistical or machine learning models for CVDys, CAS, and/or CMD diagnosis. If proven of sufficient diagnostic value, Holter monitoring at home could reduce the need for invasive testing in a subset of ANOCA patients, thereby substantially lowering the burden on patients.

## 5 Conclusion

The findings of this pilot study highlight ECG differences between ANOCA patients with and without CAS in the days preceding CFT. Patients with CAS more often demonstrated at least one minute of ST depression in total per day and exhibited periods of lower heart rates and longer PQ times mainly during the night and early morning compared to patients without CAS. Although discriminative ability was limited, we show that Holter monitoring may reveal signals in CAS patients, substantiating the need of large (AI-based) studies.

## CRediT authorship contribution statement

**Diantha JM Schipaanboord:** Writing – review & editing, Writing – original draft, Visualization, Validation, Software, Resources, Project administration, Methodology, Investigation, Formal analysis, Data curation, Conceptualization. **Caïa Crooijmans:** Writing – review & editing, Resources, Project administration, Investigation. **Nicole S Erler:** Writing – review & editing, Methodology. **Peter David Faasse:** Writing – review & editing, Project administration. **Tijn PJ Jansen:** Writing – review & editing, Resources, Project administration, Investigation. **Timo Nijkamp:** Writing – review & editing, Investigation. **Saskia ZH Rittersma:** Writing – review & editing, Supervision, Resources, Investigation, Conceptualization. **Tim P van de Hoef:** Writing – review & editing, Resources, Investigation. **Pim van der Harst:** Writing – review & editing, Supervision, Conceptualization. **Aukelien C Dimitriu-Leen:** Writing – review & editing, Resources, Investigation. **Peter Damman:** Writing – review & editing, Resources, Investigation. **Suzette E Elias-Smale:** Writing – review & editing, Supervision, Resources, Project administration. **René van Es:** Writing – review & editing, Visualization, Supervision, Software, Resources, Methodology, Conceptualization. **N Charlotte Onland-Moret:** Writing – review & editing, Visualization, Supervision, Methodology, Conceptualization. **Hester M den Ruijter:** Writing – review & editing, Visualization, Supervision, Project administration, Methodology, Funding acquisition, Conceptualization.

## Declaration of competing interest

P.D. received research grants from Abbott, Philips, AstraZeneca and Pie Medical Imaging. T.v.d.H. receives funding from the Dutch Heart Foundation (grant 030032023-0086), and has received speaker fees and institutional research grants from Abbott, Philips. ShockWave Medical, and VahatiCor. The Department of Cardiology at UMC Utrecht may receive royalties in the future from sales of deep learning ECG algorithms developed by Cordys Analytics, a spin-off company. Additionally, R.v.E. is the Chief Scientific Officer (CSO) and shareholder of Cordys Analytics. The affiliations and potential financial interests have been disclosed and are being managed in accordance with institutional policies.
