# Supplementary material for: Holter features to detect coronary artery spasm in ANOCA patients: A pilot study
Source: Int J Cardiol Heart Vasc. 2026 Feb 12;63:101884. doi: 10.1016/j.ijcha.2026.101884 (PMC12917389; doi:10.1016/j.ijcha.2026.101884)
Supplement: Supplementary Data 1 [file mmc1.docx]

**Supplementary Materials**

**Supplementary Method 1**

**Supplementary Table 1**

**Supplementary Table 2**

**Supplementary Figure 1**

**Supplementary Figure 2**

**Supplementary Figure 3**

**Supplementary Figure 4**

**Supplementary Method 1**

We filtered the raw Holter data with a third-order high-pass Butterworth filter with a cut-off frequency of 0.5 Hz. The filtered data was resampled from 200 Hz to 500 Hz and split into consecutive non-overlapping 10-second segments. We used Galvo 12L (V1.0, Cordys Analytics) to identify the locations of heartbeats and their classification, as well as the positions of P-waves, QRS complexes, ST-segments, the isoelectric line and noise artifacts. In addition, it calculates a median beat for each lead per 10-second segment. Only sinus rhythm (SR) beats were used for the analyses. All parameters were exclusively calculated if the SR beats were detected in ≥70% of the cases taking all leads combined and for segments for which the model’s output contained data on the start and end of the P-wave, start and end of the QRS complex and the end of the T-wave of at least three SR beats within the segment, where necessary for calculation.

For each 10-second segment, we calculated the median heart rate and conduction times. The corrected QT time (QTc) was calculated with Bazett’s formula. Furthermore, we calculated the standard deviation of SR beats RR intervals (SDNN; a time-domain measure of heart rate variability) per 5-minute intervals. In addition, we computed the following parameters per lead for each segment (in mV): ST deviation, positive T-wave amplitude deviation and negative T-wave amplitude deviation.

During the spasm provocation phase of the coronary function testing (CFT), the presence of ischemia is assessed by the interventional cardiologist by means of changes in the ST segment and T wave compared to a person's baseline electrocardiogram (ECG). We therefore chose to use a similar approach for the Holter analyses as the cardiologist used during CFT and examine ECG changes compared to the baseline ECG. To calculate the morphology-focused parameters, we therefore first determined a baseline 12-lead ECG of median beats per heart rate between 40-200 bpm for each patient, given the ST-segment and T-wave are influenced by heart rate. An example of such a baseline 12-lead median ECG beat for one patient for heart rates 50, 60, 70, 80, 90 and 100 beats per minute is shown in the Figure in Supplementary Method 1. To create these baseline 12-lead ECGs we first rounded the heart rates per 10-second segment. We then calculated the median of all median beats per lead for each heart rate, only if more than 100 segments of this heart rate were available and only using all symptom free segments with similar heart rates. Symptom free segments were defined as those in which patients did not report symptoms related to chest pain, pressure or tightness or shortness of breath or having difficulty breathing. In cases where symptom duration was missing, we used the mean duration of similar symptoms reported by the patient. If no similar symptoms were available, we used the mean duration of all symptoms with reported duration within a patient. In a single case, the duration from the questionnaire completed during the hospital visit was applied. Symptoms reported to persist throughout the day were assumed to last from 6:00 AM to 12:00 AM, unless a specific on- or offset time was provided. Afterwards, we performed baseline correction on the median beats by aligning the onset of the QRS complex with zero. For each lead of the baseline 12-lead ECG per heart rate, we determined its baseline ST deviation, baseline minimum T-wave amplitude and baseline maximum T-wave amplitude. ST deviation was calculated as the difference between the iso-electric line before the QRS complex and the median of the values 60 to 80 ms after the J-point, as determined by Galvo 12L. The positive and negative T-wave amplitude were determined as the maximum and minimum value within 10 ms after the J-point and 10 ms before the offset of the T-wave. To determine the ST deviation, deviation in minimum T-wave amplitude and deviation in maximum T-wave amplitude for each 10-second segment and per ECG lead, we quantified these as the difference between the median beat of the 10-second segment and the baseline median beat with corresponding heart rate.

***
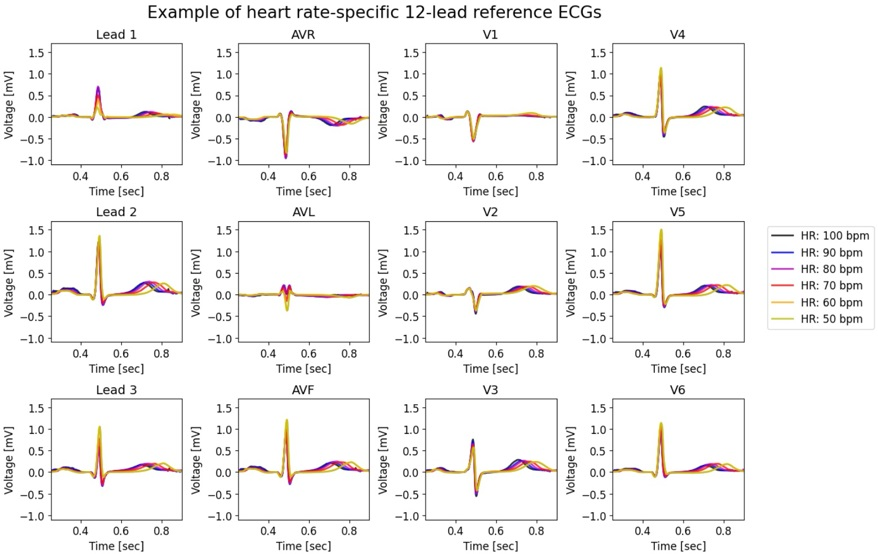
Figure of Supplementary Method 1: Example of a baseline 12-lead median ECG for heart rates 50, 60, 70, 80, 90 and 100 bpm.***

**Supplementary Table 1**

**Supplementary Table 1: Resting full-cycle ratio (RFR) and fractional flow reserve (FFR) values measured during coronary function testing, grouped by CAS diagnosis.**

|  | CAS (n=33) | No CAS (n=9) |
| --- | --- | --- |
| RFR | 0.93 [0.91-0.95]^a^ | 0.92 [0.90-0.93]^c^ |
| FFR | 0.92 [0.88-0.93]^b^ | 0.86 [0.86-0.91]^c^ |

Values are noted as median [Q1-Q3]. CAS = Coronary artery spasm.

^a^ Available from 24 participants.

^b^ Available from 30 participants*.*

^c^ Available from 8 participants.

**Supplementary Table 2**

**Supplementary Table 2: Number of patients that could be included per timepoint to compare the conduction time between those with and without CAS for A) the last full 24-hour day, and B) the first full 24-hour day.**

|  |  | **12-3 AM** | **3-6 AM** | **6-9 AM** | **9 AM-12 PM** | **12-3 PM** | **3-6 PM** | **6-9 PM** | **9 PM-12AM** |
| --- | --- | --- | --- | --- | --- | --- | --- | --- | --- |
| 1. **Last full day** | | | | | | | | | |
| **HR** | No CAS, n | 9* | 9 | 9 | 9* | 8 | 9 | 9 | 9 |
|  | CAS, n | 31* | 31 | 32 | 32* | 33 | 33 | 33 | 33 |
| **PQ** | No CAS, n | 9* | 9* | 9 | 9* | 8* | 9* | 9* | 9* |
|  | CAS, n | 31* | 31* | 32 | 32* | 33* | 33* | 33* | 33* |
| **QRS** | No CAS, n | 9* | 9* | 9* | 9* | 8* | 9* | 9* | 9* |
|  | CAS, n | 32* | 31* | 32* | 32* | 33* | 33* | 33* | 33* |
| **QT** | No CAS, n | 9 | 9 | 9* | 9* | 8 | 9 | 9* | 9 |
|  | CAS, n | 32 | 31 | 32* | 32* | 33 | 33 | 33* | 33 |
| **QTc** | No CAS, n | 9 | 9* | 9 | 9* | 8 | 9 | 9 | 9* |
|  | CAS, n | 31 | 31* | 32 | 32* | 33 | 33 | 33 | 33* |
| 1. **First full day** | | | | | | | | | |
| **HR** | No CAS, n | 9* | 9* | 9* | 9* | 8 | 9 | 9 | 9 |
|  | CAS, n | 33* | 33* | 33* | 33* | 32 | 32 | 31 | 32 |
| **PQ** | No CAS, n | 9* | 9 | 9* | 9* | 8* | 9 | 9 | 9* |
|  | CAS, n | 33* | 33 | 33* | 33* | 32* | 32 | 31 | 32* |
| **QRS** | No CAS, n | 9* | 9* | 9* | 9* | 8* | 9* | 9* | 9* |
|  | CAS, n | 33* | 33* | 33* | 33* | 32* | 32* | 31* | 32* |
| **QT** | No CAS, n | 9 | 9 | 9* | 9* | 8 | 9 | 9 | 9* |
|  | CAS, n | 33 | 33 | 33* | 33* | 32 | 32 | 31 | 32* |
| **QTc** | No CAS, n | 9 | 9* | 9 | 9 | 8* | 9* | 9 | 9 |
|  | CAS, n | 33 | 33* | 33 | 33 | 32* | 32* | 31 | 32 |

The * represents that the difference between the groups were statistically tested using a non-parametric test. CAS = Coronary artery spasm; HR = Heart rate.

**Supplementary Figure 1**


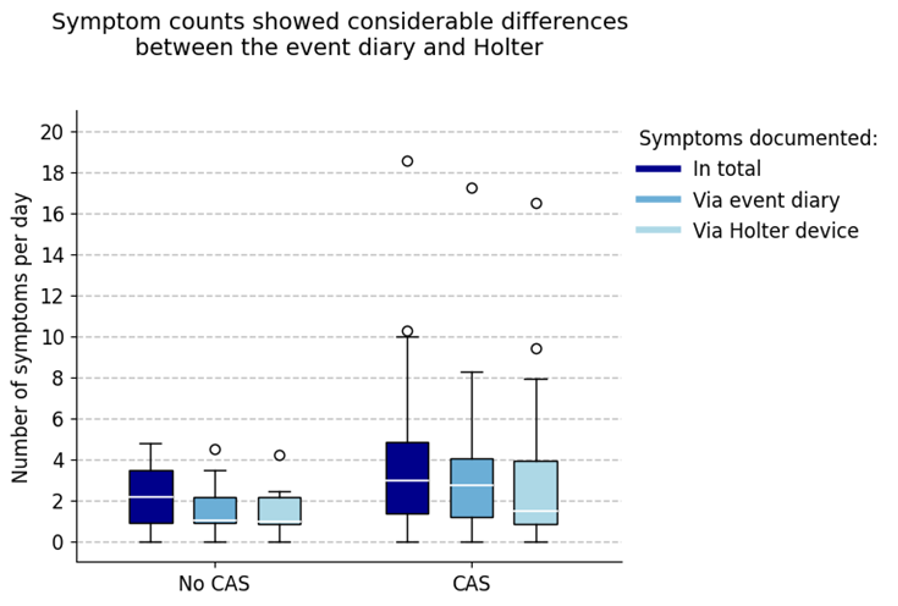
***Supplementary Figure 1: Number of symptoms per day in total (event diary and/or Holter button), via the event diary only and via the button on the Holter device only stratified by spasm provocation testing results.*** *CAS = Coronary artery spasm.*

**Supplementary Figure 2**


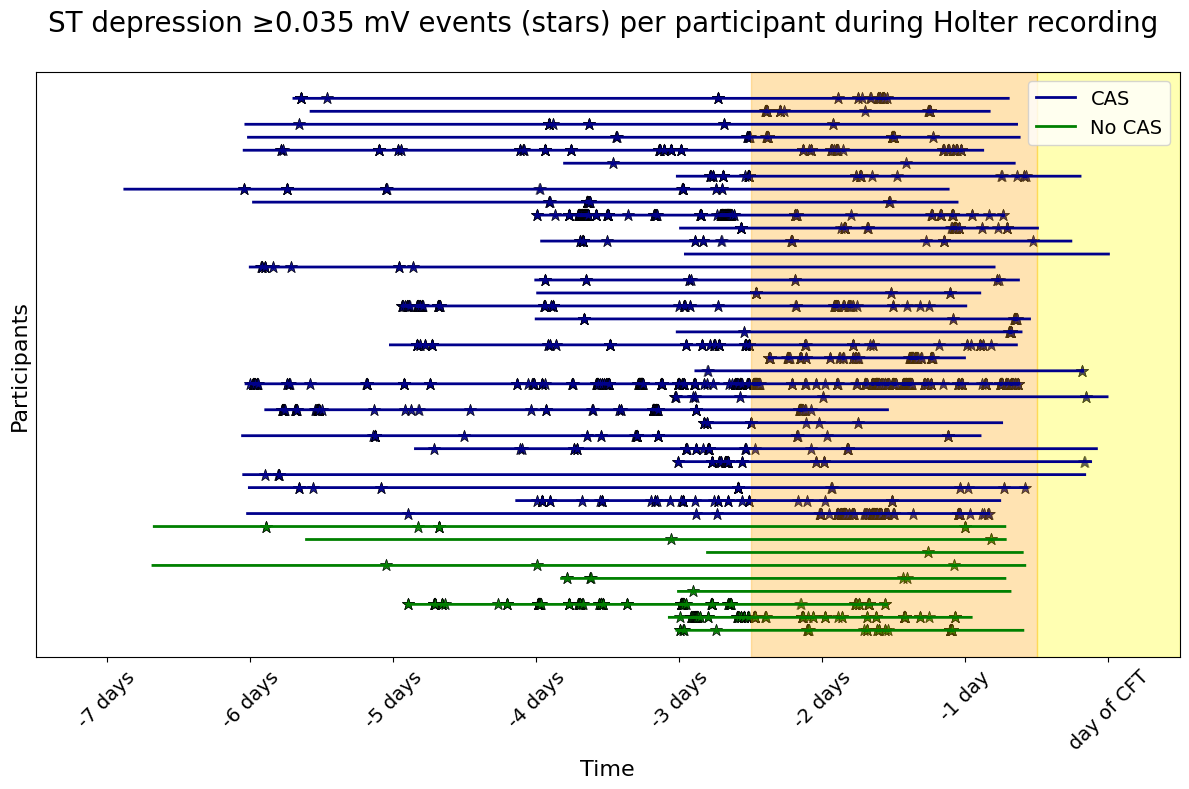


***Supplementary Figure 2: Time-dependent analysis demonstrating that the 1-2 days of medication-free period prior to CFT did not influence the occurrence of ST-segment depression.*** *For each participant, the duration of Holter monitoring is depicted as a horizontal line (blue for participants in the CAS group and green for those in the non-CAS group), with ST depression (≥0.035 mV) events indicated as stars. The yellow area represents the day of the CFT while the orange area marks the medication-free period.*

**Supplementary Figure 3**


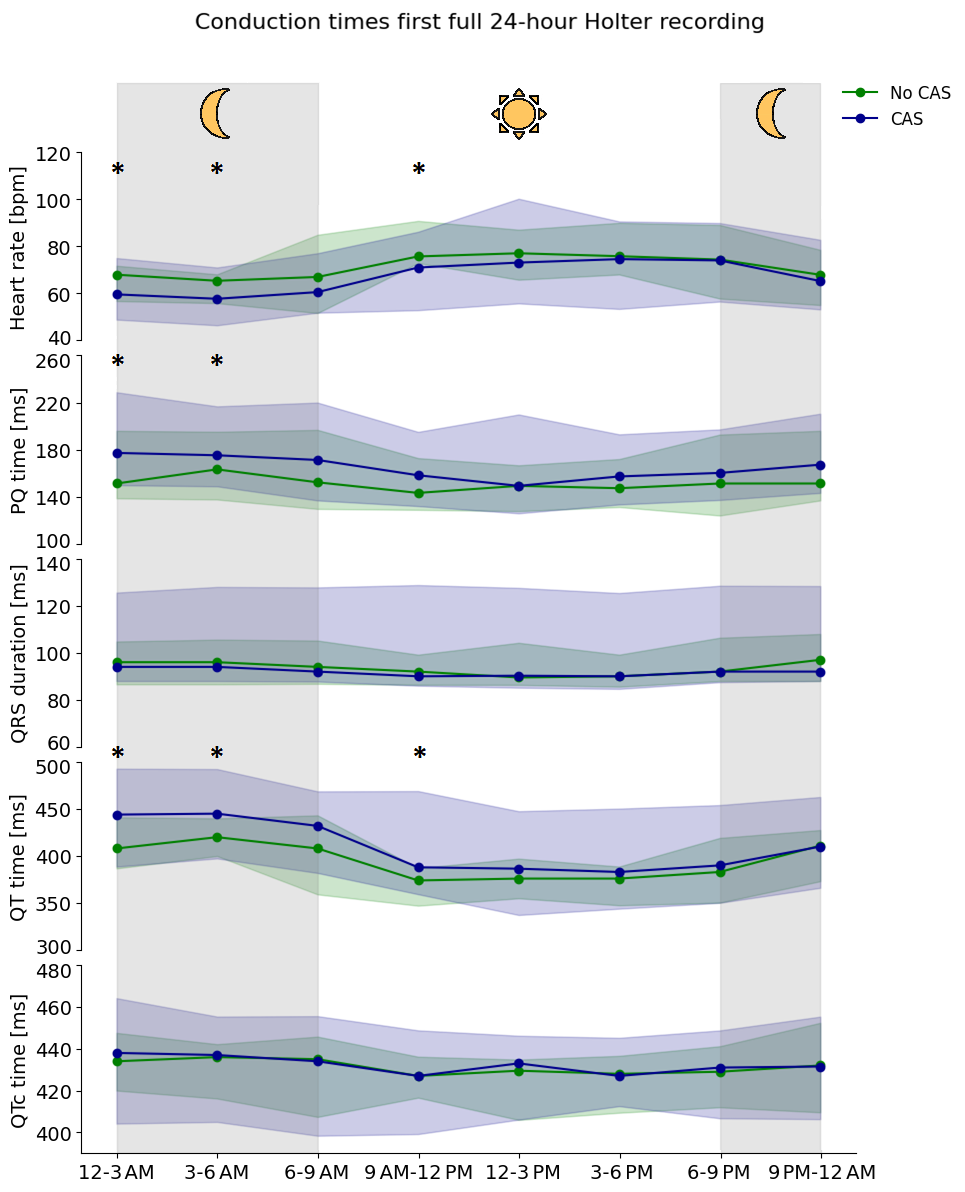


***Supplementary Figure 3: Median conduction times per 3-hours over a 24-hour period (first full day) between patients with CAS (blue) and without CAS (green).*** *The shaded colors represent the 2.5th to 97.5th percentile. The number of patients per group and type of statistical test (parametric or non-parametric) used per characteristic and timepoint is detailed in Supplementary Table 2B. The * represents a p-value<0.05.*

**Supplementary Figure 4**


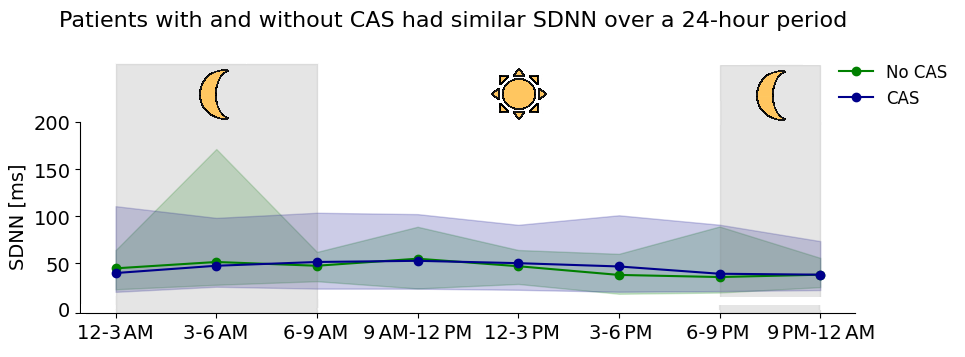
***Supplementary Figure 4: Median of the standard deviation of intervals between successive normal heartbeats (SDNN) per 3-hours over a 24-hour period (last full day) between patients with CAS (blue) and without CAS (green).*** *The shaded colors represent the 2.5th to 97.5th percentile.*
